# Signatures of selection for resistance to *Haemonchus contortus* in sheep and goats

**DOI:** 10.1186/s12864-019-6150-y

**Published:** 2019-10-15

**Authors:** Zaira M. Estrada-Reyes, Yoko Tsukahara, Rodrigo R. Amadeu, Arthur L. Goetsch, Terry A. Gipson, Tilahun Sahlu, Richard Puchala, Zaisen Wang, Steve P. Hart, Raluca G. Mateescu

**Affiliations:** 1 Department of Animal Sciences, University of Florida, Gainesville, FL USA; 2 American Institute for Goat Research, Langston University, Langston, OK USA; 3 Horticultural Sciences Department, University of Florida, Gainesville, FL USA

**Keywords:** *F*st, *Haemonchus contortus*, Signatures of selection, SNP, Sheep, Goats, Immune response

## Abstract

**Background:**

Gastrointestinal nematode infection (GNI) is the most important disease affecting the small ruminant industry in U.S. The environmental conditions in the southern United States are ideal for the survival of the most pathogenic gastrointestinal nematode, *Haemonchus contortus*. Host genetic variation for resistance to *H. contortus* allows selective breeding for increased resistance of animals. This selection process increases the prevalence of particular alleles in sheep and goats and creates unique genetic patterns in the genome of these species. The aim of this study was to identify loci with divergent allelic frequencies in a candidate gene panel of 100 genes using two different approaches (frequentist and Bayesian) to estimate *F*st outliers in three different breeds of sheep and goats exposed to *H. contortus*.

**Results:**

Our results for sheep populations showed SNPs under selection in *C3AR1*, *CSF3*, *SOCS2*, *NOS2*, *STAT5B*, *TGFB2* and *IL2RA* genes using frequentist and Bayesian approaches. For goats, SNPs in *CD1D*, *ITGA9*, *IL12A*, *IL13RA1, CD86* and *TGFB2* genes were under selection. Common signatures of selection in both species were observed in *NOS2*, *TGFB2* and *TLR4* genes. Directional selection was present in all SNPs evaluated in the present study.

**Conclusions:**

A total of 13 SNPs within 7 genes of our candidate gene panel related to *H. contortus* exposure were identified under selection in sheep populations. For goats, 11 SNPs within 7 genes were identified under selection. Results from this study support the hypothesis that resistance to *H. contortus* is likely to be controlled by many loci. Shared signatures of selection related to mechanisms of immune protection against *H. contortus* infection in sheep and goats could be useful targets in breeding programs aimed to produce resistant animals with low FEC.

## Background

Small ruminant industry in the US is a growing industry due to ethnic markets and increasing demand for organically produced livestock. Gastrointestinal nematode infection (GNI) is one of the most prevalent health problems in sheep and goats and represents a major productivity threat for small ruminants [1]. High disease incidence has been observed in the Southeast US regions [2] and infection with *Haemonchus contortus* is common throughout the year [3, 4]. This blood sucking parasite inhabits the abomasum of the host and it is responsible for weight loss, anemia and reduced performance [2].

Recent advances in genomic research have provided tools to unravel the genetics underlying phenotypic variation in complex traits [5], including resistance to GNI. Host genetic variation for GNI promises great opportunities for selective breeding of sheep and goats with increased resistance to *H. contortus*. Fecal egg count (FEC) is currently the method of choice to identify resistance to GNI and is the standard phenotypic measure to achieve rapid genetic progress [6]. Host resistance based on FEC is a heritable trait in both sheep and goats, with heritability estimates ranging between 0.01 to 0.65, and 0.1 to 0.33, respectively [7–16]. In accordance, breeding studies of small ruminants have revealed a FEC reduction after concurrent selective breeding of naturally resistant sheep to GIN infection [17–19].

Sheep and goats were the first livestock species to be domesticated by humans and were initially used mainly for meat, rather than wool or milk [20]. Natural selection and artificial selective breeding are the main driving forces shaping genetic variation across the sheep and goat genomes, and have gradually changed the phenotypes of these species. Within breeding strategies, selection increases the frequency of particular alleles at different loci in the population and creates unique genetic patterns in the DNA sequence that can be traced back and investigated for further analyses [21].

Two of the most used statistical methods for the analysis of signatures of selection are the detection of long haplotypes and the identification of differences in allele frequencies. The long haplotype detection method requires accurate allele assignment to one of the parental chromosomes (chromosome phasing) and ancestral allele identification, which sometimes can be a limitation when information about ancestors and pedigree is not available [22]. On the other hand, genetic differentiation among groups can be computed using the *F*st method. This approach allows for identification of loci showing differences in allelic frequencies between two or more divergent populations, and therefore believed to be under selection. Highly genetic divergent loci between populations have more extreme *F*st values (greater than 0.25) than low genetic divergent loci [23], and extreme *F*st values are associated with either natural or artificial selection.

Using this approach for sheep, few loci have been identified as regions under selection for resistance or susceptibility to GNI [24], and in goats, information is even more scarce [25]. Some candidate markers within *Ovar-DRA* and *Ovar-DRB* genes have been identified as possible genetic markers associated with low *H. contortus* FEC in sheep and goat populations [26]. However, more knowledge is required to understand the genetic architecture underlying resistance against GNI in these species. Thus, the aim of this study was to identify immune loci (among a candidate gene panel of 100 genes) with divergent allelic frequencies in three different lines of sheep and goats, respectively, using the *F*st statistic.

## Results

### Genotyping, quality control and population structure in sheep and goats

The sheep and goat DNA samples were sequenced with a median depth of 24x across 5000 probes (average of 50 probes/gene) The initial SNP data set consisted of 5346 SNPs for both sheep and goats. Only biallelic SNPs were identified in our populations. After quality control, the final SNP data set contained 1339 SNPs for sheep and 1020 SNPs for goats.

**Fig. 1:**
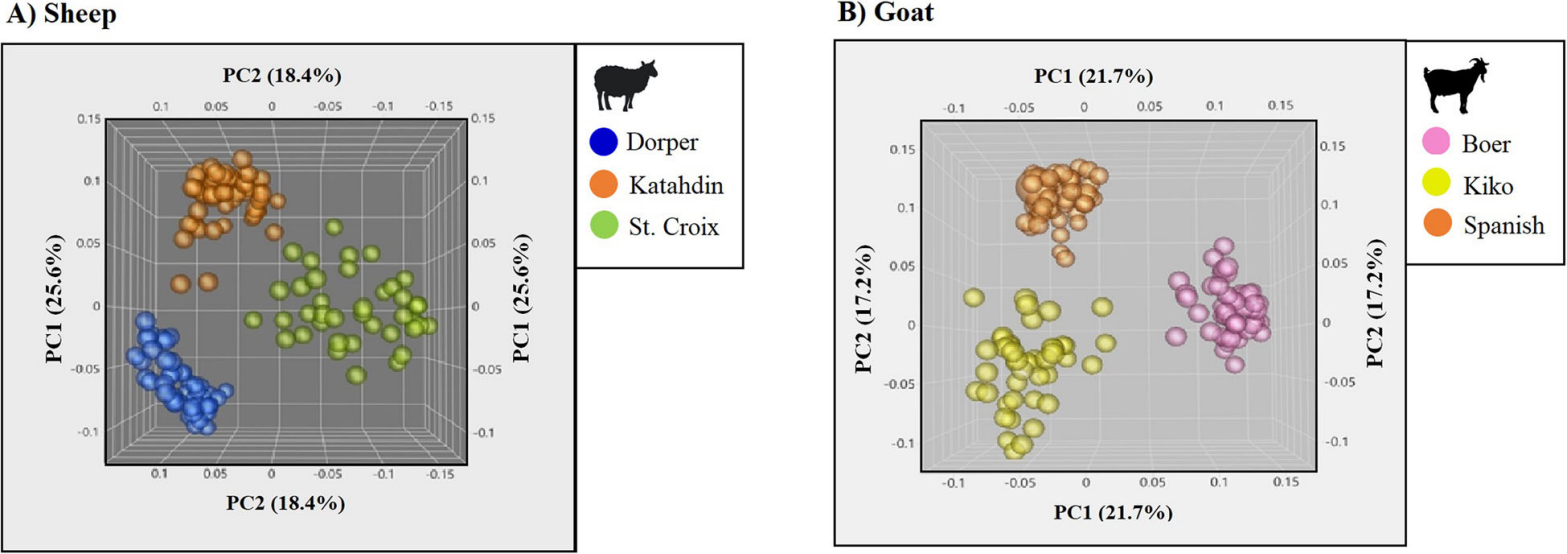
Sheep and goat PCA plots. The PCA plots show the population structure of the breeds under the study based on the first two principal components (PC). For sheep (**a**), the PC1 and PC2 explain 25.6 and 18.4% of the total variation, respectively. For goats (**b**), the PC1 and PC2 explain 21.7 and 17.2% of the total variation observed in the populations, respectively

The plot from principal component analysis (PCA) for sheep (Fig. 1a), presented one specific cluster per breed. The first two principal components explained 25.6 and 18.4% of the total variance observed in sheep, respectively. For goats, the PCA plot (Fig. 1b) clustered the animals within breed and one specific cluster was observed per breed. PC1 and PC2 explained 21.7 and 17.2% of the total variance observed in goats, respectively.

### FEC descriptive statistics in sheep and goats

Dorper sheep had the highest FEC (1475 ± 207.4 eggs per gram of feces) across breeds with Katahdin (1087 ± 191.2) and St. Croix (969 ± 180.6) sheep, which were considered resistant in this study, presenting lower FEC. Thus, Katahdin and St. Croix breeds had 26.3 and 34.3% less eggs per gram of feces than Dorper sheep, respectively.

For goats, Boer goats had 1548 ± 173.1 eggs per gram of feces. Kiko (936 ± 159.1) and Spanish (911 ± 150.9), categorized as resistant breeds, had 39.5 and 41.1% less eggs per gram of feces than Boer goats, respectively.

### Signatures of selection using *F*st in sheep

**Fig. 2:**
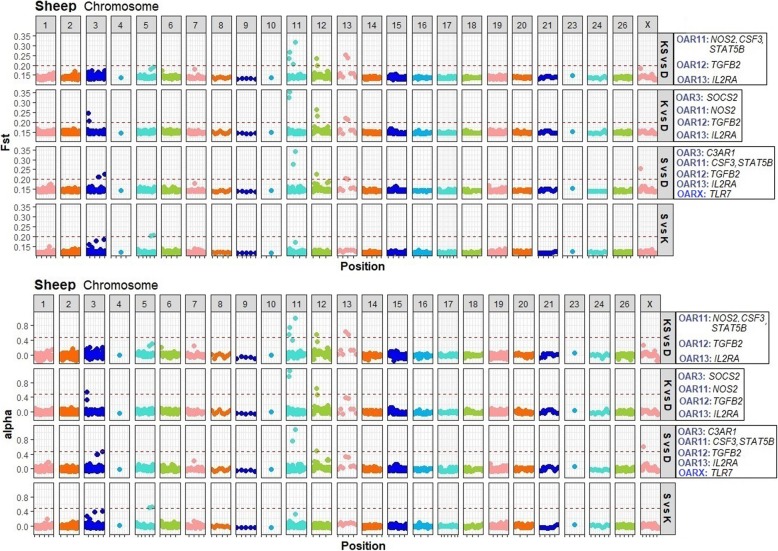
Manhattan plots of the Bayesian *F*st values per SNP from targeted genomic regions in sheep. The SNP data is ordered based on chromosomal location (x-axis). The plots includes: Katahdin and St. Croix vs Dorper (KS vs D, pooled resistant breeds vs susceptible breed), Katahdin vs Dorper (K vs D, resistant vs susceptible breed), St. Croix vs Dorper (S vs D, resistant vs susceptible breed) and St. Croix vs Katahdin (S vs K, resistant vs resistant breed) analyses. The genes with SNPs under selection in each analysis are presented in a box on the right side of Manhattan plots

A total of 18 different SNPs in *CCR3, CD86, EPS15, TLR4, IL2RB, STAT2, C3AR1, SOCS2, TLR10, NOS2, CSF3, STAT5B, TGFB2, LAMC1, IL2RA* and *IL12RA1* genes were identified under selection using the frequentist *F*st (Additional file 1: Figure S1, Additional file 2: Table S1). For Bayesian *F*st, 14 SNPs were observed under selection in *C3AR1, LTBR, SOCS2, CSF3, NOS2, STAT5B, TGFB2, IL2RA*, and *TLR7* genes (Figure 2, Additional file 3: Table S2). Using this approach, *F*st values greater than 0.20 were observed in the sheep populations and within OAR 3, 11, 12, 13 and X. The sign of alpha was always positive which indicates that, in all cases, directional selection was present in the SNPs under selection.

**Fig. 3:**
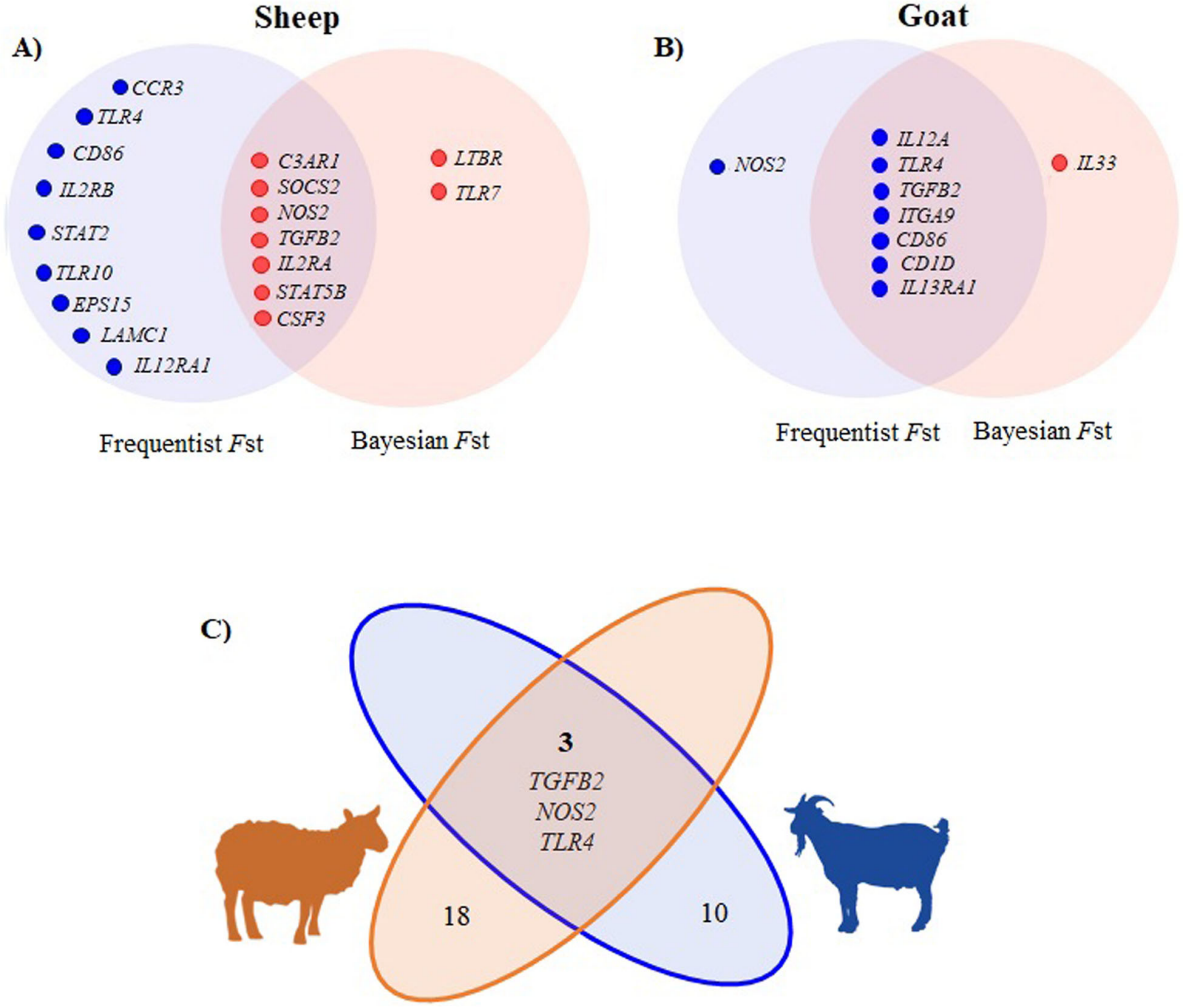
Venn diagram of the shared signatures of selection between frequentist and Bayesian *F*st for sheep (**a**) and goats (**b**) and the common signatures of selection in both species (**c**)

Shared signatures of selection between both approaches were identified in *C3AR1, SOCS2, NOS2, CSF3, TGFB2, IL2RA* and *STAT5B* genes (Table 1 and Figure 3). For Katahdin and St. Croix vs Dorper analysis (Table 1), OAR 11 and 12 and 13 contained extreme *F*st values in *CSF3*, *NOS2*, *TGFB2* and *IL2RA* genes. The *NOS2* and *IL2RA* genes were the genomic regions with most loci under selection. The SNPs (OAR11:18963484 and OAR11:18963494) within exon 7 and 16 of *NOS2* gene (A/G and T/C) are synonymous mutations.

**Table 1:** Signatures of selection identified between resistant (Katahdin or St. Croix) and susceptible (Dorper) sheep breeds. Breeds compared (comparison), gene name, gene region, SNP name (chromosome and position), SNP, mutation type (synonymous or missense), and *F*st value for the SNPs under selection

Comparison	Gene	Region	SNP name	SNP	MAF	Mutation	*F*st (Bayesian)	*F*st (Frequentist)	alpha
Katahdin and St. Croix vs Dorper (Resistant and Resistant vs Susceptible)	CSF3	5’UTR	OAR11:39857496	G/A	Dorper: 0.20, Katahdin: 0.19, Spanish: 0.35		0.31	0.35	1
	NOS2	Exon 7	OAR11:18963484	A/G	Dorper: 0.21, Katahdin: 0.19, Spanish: 0.23	Synonymous (Ile → Ile)	0.36	0.35	0.67
	NOS2	Exon 16	OAR11:18963494	T/C	Dorper: 0.20, Katahdin: 0.19, Spanish: 0.25	Synonymous (Leu → Leu)	0.33	0.35	0.55
	TGFB2	3’UTR	OAR12:19965761	A/C	Dorper: 0.20, Katahdin: 0.12, Spanish: 0.16		0.26	0.57	0.52
	IL2RA	Intron 5	OAR13:10442920	C/A	Dorper: 0.55, Katahdin: 0, Spanish: 0		0.24	0.21	0.6
	IL2RA	Intron 5	OAR13:10442953	A/G	Dorper: 0.43, Katahdin: 0, Spanish: 0		0.23	0.21	0.55
Katahdin vs Dorper (Resistant vs Susceptible)	SOCS2	Exon 2	OAR3:129558034	C/T	Dorper: 0.20, Katahdin: 0.19	Synonymous (Ile → Ile)	0.25	0.6	0.53
	SOCS2	3′ UTR	OAR3:129558430	G/A	Dorper: 0.20, Katahdin: 0.19		0.21	0.56	0.39
	NOS2	Exon 7	OAR11:18963484	A/G	Dorper: 0.21, Katahdin: 0.19	Synonymous (Ile → Ile)	0.36	0.59	1
	NOS2	Exon 16	OAR11:18963494	T/C	Dorper: 0.20, Katahdin: 0.19	Synonymous (Leu → Leu)	0.33	0.56	0.97
	TGFB2	3’UTR	OAR12:19965761	A/C	Dorper: 0.20, Katahdin: 0.12		0.26	0.57	0.6
	TGFB2	3’UTR	OAR12:19965865	A/C	Dorper: 0.05, Katahdin: 0.29		0.25	0.51	0.5
	IL2RA	Intron 5	OAR13:10442920	C/A	Dorper: 0.55, Katahdin: 0		0.24	0.21	0.42
	IL2RA	Intron 5	OAR13:10442953	A/G	Dorper: 0.43, Katahdin: 0		0.23	0.22	0.39
St. Croix vs Dorper (Resistant vs Susceptible)	C3AR1	3’UTR	OAR3:206099209	T/A	Dorper: 0, Spanish: 0.42		0.21	0.35	0.52
	CSF3	5’UTR	OAR11:39857496	G/A	Dorper: 0.20, Spanish: 0.35		0.34	0.48	1
	STAT5B	Intron 16	OAR11:41755713	G/A	Dorper: 0.20, Spanish: 0.48		0.28	0.36	0.76
	TGFB2	3’UTR	OAR12:19965761	A/C	Dorper: 0.20, Spanish: 0.16		0.23	0.55	0.49
	IL2RA	Intron 5	OAR13:10442920	C/A	Dorper: 0.55, Spanish: 0		0.21	0.21	0.4

For the Katahdin vs Dorper analysis (Table 1), 8 SNPs under selection were observed in *SOCS2*, *NOS2*, *TGFB2* and *IL2RA* genes within OAR3, 11, 12 and 13, respectively. The SNPs in *NOS2*, *TGFB2*, and *IL2RA* genes were also observed with high genetic differentiation in the Katahdin and St. Croix vs Dorper analysis. The OAR3:129558034 (*SOCS2*), OAR11:18963484 (*NOS2*), and OAR11:18963494 (*NOS2*) are synonymous mutations.

For the St. Croix vs Dorper analysis (Table 1), 5 SNPs within OAR 3, 11, 12 and 13 were observed under selection in untranslated and intronic regions. The genes showing high genetic differentiation were *C3AR1, CSF3, STAT5B*, *TGFB2*, and *IL2RA*. The highest *F*st value (0.34 for BayeScan and 0.48 for R software) was found in a SNP (OAR3:206099209) located in the 5’UTR region of *CSF3*gene. The SNPs (OAR11:39857496, OAR12:19965761, and OAR13:10442920) in *CSF3*, *TGFB2* and *IL2RA* genes were also observed under selection in the Katahdin and St. Croix vs Dorper analysis.

### Signatures of selection using *F*st in goats

**Fig. 4:**
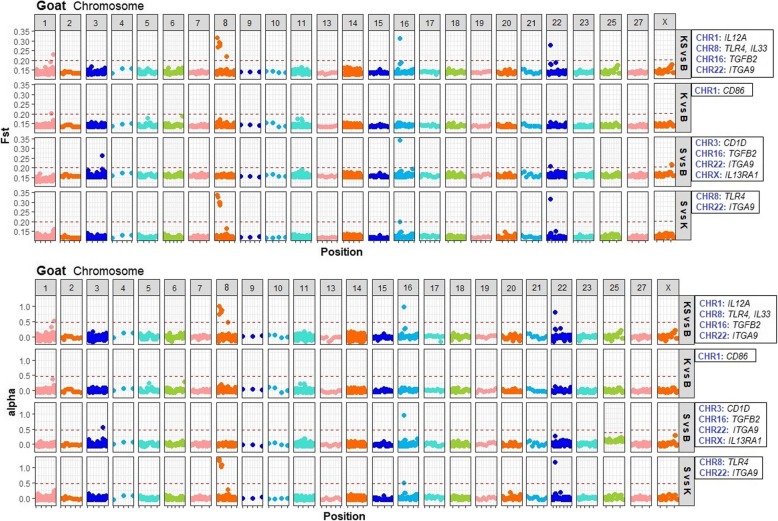
Manhattan plots of the Bayesian *F*st values per SNP from targeted genomic regions in goats. The SNP data is ordered based on chromosomal location (x-axis). The plots includes: Kiko and Spanish vs Boer (KS vs B, pooled resistant breeds vs susceptible breed), Kiko vs Boer (K vs B, resistant vs susceptible breed), Spanish vs Boer (S vs B, resistant vs susceptible breed) and Spanish vs Kiko (S vs K, resistant vs resistant breed) analyses. The genes with SNPs under selection in each analysis are presented in a box on the right side of Manhattan plots

For goats, genes within CHR 1, 3, 8, 16, 19 and 22 contained 13 SNPs under selection using the frequentist *F*st (Additional file 4: Table S3, Additional file 1: Fig. S1). Using this approach, genes with loci under selection in goat populations were *IL12A, TLR4*, *TGFB2*, *ITGA9*, *CD86*, *CD1D*, *NOS2*, and *IL13RA1*. Signatures of selection detected with the Bayesian *F*st were identified in 11 SNPs harboring *IL12A, TLR4*, *IL33*, *TGFB2*, *ITGA9*, *CD86*, *CD1D*, and *IL13RA1* genes (Additional file 5: Table S4, Figure 4). All the SNPs were under directional selection and located in exonic, intronic and untranslated regions (Figure 4).

Information regarding shared SNPs under selection in goats using both frequentist and Bayesian approaches is presented in Table 2 and Figure 3. For the Kiko and Spanish vs Boer analysis (Table 2), *IL12A*, *TLR4* and *ITGA9* genes had 4 SNPs under selection. The highest *F*st value was observed in the CHR8:106725462 (*TLR4*). The CHR8:106725462 and CHR8:106725265 in exon 3 of *TLR4* gene are synonymous mutations.

**Table 2:** Signatures of selection identified between resistant (Kiko or Spanish) and susceptible (Boer) goat breeds. Breeds compared (comparison), gene name, gene region, SNP name (chromosome and position), SNP, mutation type (synonymous or missense), and *F*st value for the SNPs under selection

Comparison	Gene	Region	SNP name	SNP	MAF across breeds	Mutation	*F*st (Bayesian)	*F*st (Frequentist)	alpha
Kiko and Spanish vs Boer (Resistant and Resistant vs Susceptible)	IL12A	5’UTR	CHR1: 106973801	A/G	Boer: 0, Kiko: 0.3, Spanish:0		0.23	0.4	0.55
	TLR4	Exon 3	CHR8:106725462	T/C	Boer: 0.05, Kiko: 0.36, Spanish: 0	Synonymous (Ser → Ser)	0.30	0.36	0.91
	TLR4	Exon 3	CHR8:106725265	A/G	Boer: 0.05, Kiko: 0.3, Spanish: 0	Synonymous (Leu → Leu)	0.28	0.35	1
	ITGA9	3’UTR	CHR22:11106216	A/T	Boer: 0.48, Kiko: 0.42, Spanish: 0		0.28	0.36	0.75
Kiko vs Boer (Resistant vs Susceptible)	CD86	Intron 1	CHR1:66217253	C/T	Boer: 0, Kiko: 0.15		0.21	0.54	0.46
Spanish vs Boer (Resistant vs Susceptible)	CD1D	Exon 2	CHR3:107890049	T/G	Boer: 0.20, Spanish: 0.28	Synonymous (Ser → Ser)	0.27	0.61	0.57
	TGFB2	3’UTR	CHR16:20438403	T/G	Boer: 0, Spanish: 0.38		0.35	0.22	0.98
	ITGA9	3’UTR	CHR22:11106216	A/T	Boer: 0.48, Spanish: 0		0.21	0.37	0.3
	IL13RA1	3’UTR	CHRX:25115674	G/T	Boer: 0, Spanish: 0.27		0.22	0.21	0.3
Spanish vs Kiko (Resistant vs Resistant)	TLR4	Exon 3	CHR8:106725462	T/C	Kiko: 0.36, Spanish: 0	Synonymous (Ser → Ser)	0.3	0.45	1
	TLR4	Exon 3	CHR8:106725265	A/G	Kiko: 0.3, Spanish: 0	Synonymous (Leu → Leu)	0.29	0.45	1
	TLR4	Exon 4	CHR8:106725156	G/A	Kiko: 0.32, Spanish:0	Synonymous (His → His)	0.29	0.44	0.99
	TLR4	Exon 4	CHR8:106725045	C/T	Kiko: 0.34, Spanish:0	Synonymous (Leu → Leu)	0.29	0.43	0.99
	ITGA9	3’UTR	CHR22:11106216	A/T	Kiko: 0.42, Spanish: 0		0.32	0.35	1

For the Kiko vs Boer analysis (Table 2), high genetic differentiation was observed in a SNP (CHR1:66217253) located in an intronic region of *CD86* gene. For the Spanish vs Boer analysis (Table 2), *CD1D*, *TGFB2*, *ITGA9* and *IL13RA1* genes contained 4 SNPs under selection. The SNP (T/G) observed under selection in exon 2 of *CD1D* gene generates a synonymous mutation. Loci under selection in *TGFB2*, *ITGA9* and *IL13RA1* genes were identified in the untranslated regions.

Finally, for the Spanish vs Kiko analysis (Table 2), 5 SNPs within *TLR4*, and *ITGA9* genes were observed under selection. The majority of the SNPs with divergent allelic frequencies were located in *TLR4* gene and within exon 3 and 4. Two (CHR8:106725462 and CHR8:106725265) out of 4 of the SNPs in *TLR4* gene were also observed under selection in the Kiko and Spanish vs Boer analysis. The CHR22:11106216 was also under directional selection in the Kiko and Spanish vs Boer analysis, and in the Spanish vs Boer analysis.

### Genomic regions under selection in both species

After examination of the *F*st results per species, several loci in *TGFB2*, *NOS2*, and *TLR4* genes were observed under selection in both species and are presented in Table 3 and Figure 3.

**Table 3:** Common signatures of selection identified in sheep and goats. Sheep chromosome (OAR), goat chromosome (CHR), gene name, and gene cellular function

OAR	CHR	Gene	Function
12	16	TGFB2	Regulation of gene expression
11	19	NOS2	Synthesis of nitric oxide/ regulator of macrophage functions
2	8	TLR4	Cell activation

## Discussion

Domestication, breed formation, and selective breeding leave detectable patterns within the genome of livestock species such as sheep and goats. Identification of these genomic patterns in the DNA sequence could help to identify of genes controlling resistance to *H. contortus* or other gastrointestinal parasites. Several studies have attempted to identify the genetic variation controlling gastrointestinal parasite resistance in sheep and goats by using SNP markers and Genome Wide Association Studies (GWAS) but few research studies has been devoted to identify signatures of selection for GNI resistance in these species [27–29]. Signatures of selection for resistance to GNI have not been identified in goats, and for sheep, only Perendale and Romney breeds have been evaluated [24]. In the present study, we unravel signatures of selection using a targeted sequencing approach in three different breeds of sheep and goats. The SNPs potentially under selection identified in this study spanned a myriad of candidate genes related to immune response and cellular mechanisms against *H. contortus*.

In our study, all the signatures of selection identified in sheep and goats were under directional selection. Directional selection is one of the primary cause of phenotypic diversification and has been used to increase the frequency of favorable additive alleles [30]. This selection process has not exhausted the genetic variation for most economically important traits in livestock [31]. Our results suggest that some of the SNPs in genes related to resistance to GNI are under directional selection. This could be possible due to selection for resistance to GNI is focused on resistant individuals, and susceptible animals are usually removed from the flock or not used in the mating process.

### Signatures of selection in sheep populations

For Katahdin vs Dorper, and Katahdin and St. Croix vs Dorper analyses, loci within *SOCS2*, *NOS2*, *TGFB2* and *IL2RA* genes were observed under selection. The *SOCS2* gene has been previously associated with FEC in Dorper x Red Maasai sheep using GWAS and expression of this candidate gene was observed in abomasal tissue, mesenteric lymph nodes, and Peyer’s patches from ewes and lambs [27]. Thus, it is possible that *SOCS2* gene could be used as candidate gene for future studies to validate previous and current results in Dorper and Dorper × Red Maasai sheep.

The *SOCS2* gene is a broad key regulator of cytokine responses, including IL2, IL3, IL4, IFN-γ, CSF, and Jak-STAT signaling pathways in bone marrow and T cells [32]. Studies on mice infected with *Tripanozoma cruzi* have shown that expression of *SOCS2* facilitates inflammatory and immune responses to prevent myocardial dysfunction but increases parasitemia [33]. On the contrary, *SOCS2*^−/−^ mice infected with *Schistosoma mansoni* expressed increased Th2 response with higher IgE and eosinophil production than *SOCS2*^+/+^ mice [34]. Also, SOCS2^−/−^ mice have shown increased body weight and gigantism possibly due to downregulation of growth hormone and insulin-like growth factor-I (IGF-I) signaling [35]. In Scottish Blackface sheep infected with *Teladorsagia circumcincta*, *SOCS2* gene was found differentially expressed between resistant and control animals [36].

Nitrogen oxygen synthase 2 or *NOS2* is a key molecule involved in Th1 response. It participates in the production of nitric oxide to kill invading microbes in phagocytes during classical macrophage activation by IFN-γ and TNF-α. Differential expression of this gene has been observed in the abomasum of Merino sheep during *H. contortus* challenge [37]. In that study, mRNA expression *NOS2* gene was downregulated in susceptible individuals. While there is a proposed interplay between Th1, Th2, and Treg responses during GNI [38], susceptibility to these infections has been related to Th1 and Th17 responses, and Th2 has been associated with resistance to helminth infections in sheep [39].

TGFB2 protein has been reported as an anti-inflammatory cytokine, and was observed in high concentration in the gut mucosa of sheep after *H. contortus* infection [40]. In pigs, PAS1, a product of *Ascaris suum,* induces IL-10 and TGFB2 production in macrophages and has been related to loss of pro-inflammatory cytokine production [41]. In humans and animal models, it has been shown that inhibition of T-cell proliferation might be triggered by an increase of IL-10 and TGFB production in antigen presenting cells or T-cells as a result of down-modulatory molecules that are released by the parasites to enhance survival [42]. Thus, parasites are prone to use IL-10 and TGFB to downregulate host immune response.

IL2RA protein is mainly expressed in CD4+ Treg cells and it constitutes one of the three subunits of the IL2R. In humans, induction of Treg cells increases during natural and long term gastrointestinal nematode infections [43, 44]. In sheep, expression of the mRNA of *IL2RA* gene in the abomasum has been related to subsequent *H. contortus* infections in resistant sheep while its expression in the jejunal mucosa has been linked to susceptibility of *Trichostrongylus columbriformis* [37]. Thus, differential expression between susceptible and resistant individuals could depend on the stage of the host immune response, the infection period, as well as the nematode species.

For St. Croix vs Dorper, and Katahdin and St. Croix vs Dorper analyses, SNPs in *C3AR1*, *CSF3*, *STAT5B*, *TGFB2* and *IL2RA* genes were found to be under selection. C3AR1 protein plays an important role during innate immune responses. It is part of the complement cascade. Reduced T cell responses has been observed when host animals lack of C3AR [45]. Recent work in mice, using bone marrow transplant and RNA Seq analysis, identified that signaling by C3AR mediates macrophage recruitment after induced injury with cardiotoxin and muscle regeneration [46]. The exact role of C3AR1 has not been evaluated in sheep during gastrointestinal nematode infections, but some studies suggest that the complement activation is one of the first mechanisms of protection against helminth infections [47] and classical and alternative complement pathways can be activated in resistant sheep to *H. contortus* [48].

The *STAT5* gene can be activated by many cytokines such as GM-CSF and thymic stromal lymphopoietin (TSLP) in the dendritic cells. Activation of *STAT5* by TSLP has been shown to trigger Th2 responses at barrier surfaces [49]. Also, *STAT5* signaling has been related to many biological processes, such as TCR signaling and basal proliferation of naïve CD4+ T cells [50]. Moreover, STAT5B mediates the signal transduction of IL2, IL4, CSF1, and different growth hormones. Thus, it is possible that *STAT5B* gene is responsible for many cellular functions during *H. contortus* exposure and further analysis is required to confirm our results.

### Signatures of selection in goat populations

For many years, there has been a debate about the immune mechanisms controlling *H. contortus* infections in sheep and goats. The same helminth species can parasitize both species but previous studies suggest higher levels of infection in goats [51].

For Kiko and Spanish vs Boer, genes with genetic differentiation were *IL12A*, *TLR4* and *ITGA9*. IL12 protein is a major cytokine that controls the maturation of CD4+ T cells into Th1 cells and promotes IFNG production in response to intracellular parasites. IL12 protein is composed of an alpha chain (p35 subunit) and a beta chain (p40 subunit) linked by a disulfide bond [52]. In Nelore cattle, some studies have suggested that susceptibility to gastrointestinal parasites is associated with an increase of Th1 response with high elevated worm burden and elevated IFNG and IL12 production [53].

Toll-like receptors (TLR) are vital for the detection of invading pathogens and are commonly expressed in antigen presenting cells and other immune cells [54]. In resistant sheep infected with *H. contortus* and *T. colubriformis*, upregulation of several *TLR* genes, including *TLR*4, was observed in the abomasum. In the same study, susceptible individuals presented lower expression of this gene [37]. Contrary to sheep, susceptible Angus yearlings infected with *Ostertagia*, *Cooperia* and *Nematodirus* spp., *TLR4* showed higher expression in the mesenteric lymph nodes [55]. In goats, increased expression of *TLR4* gene in blood has been observed during inclusion of *Sericea lespedeza* in the diet [56]. This observation could be related to biologically active tannin fractions from plants containing tannins such as *S. lespedeza.* Several studies have shown plant tannins are able to modulate the innate immune response and act as γ-T cell agonists [56, 57].

The *ITGA9* gene encodes an alpha integrin that compose the integral membrane glycoproteins that mediates cell-cell and cell matrix adhesion. In resistant Merino sheep infected with *H. contortus*, transcriptome analysis results revealed *ITGA9* gene as part of an enriched gene set related to the extracellular matrix receptor interaction pathway [58]. The exact role of *ITGA9* gene in goats is unknown, but further analysis could help to understand possible mechanisms of protection against *H. contortus* and other gastrointestinal parasites.

For Kiko vs Boer, a SNP in *CD86* gene was observed under selection. This gene encodes a membrane bound protein in antigen presenting cells that binds CD28 and CTLA-4 proteins localized in the T cell membrane. Binding with C28 leads to T cell proliferation and cytokine production, while binding with CTLA-4 negatively regulates T cell response [59]. Thus, it is possible that CD86 controls some T cell mechanisms in goats.

For Spanish vs Boer analysis, signatures of selection were identified in *CD1D*, *TGFB2*, *ITGA9* and *IL13RA1* genes. CD1D is a major histocompatibility complex class I related protein that regulates presentation of glycolipids antigens to natural killer T cells [60]. In resistant cattle naturally exposed to gastrointestinal parasites, CD1D was upregulated in the mesenteric lymph nodes [55]. In goats, the role of CD1D has not been studied but it is possible that this gene could play an important role during presentation of glycoproteins from *H. contortus* to T cells.

As observed in sheep, TGFB2 could be used by *H. contortus* to promote infection. In tropical cattle, susceptibility to *Theileria annulata* has been associated with TGFB2 induction and increased TGF-b2 production by *Theileria*-infected macrophages promote invasiveness [61]. In sheep, TGFB-like molecules have been identified in larvae from *H. contortus* and *T. circumcincta* [62]. Thus, activation of TGFB and TGFB-like molecules from gastrointestinal parasites could control downregulation of the immune response. The exact role of TGFB2 in goats is unclear and more research is needed to understand its function during *H. contortus* infections.

IL13RA1 subunit together with IL4RA can form a functional receptor for IL13 [63]. In goats, no evidence of IL13RA1 function has been reported but in Hereford Shorthorn cattle infected with *Boophilus microplus*, results showed that *IL13RA1* precursor was differentially expressed [64].

Finally for Spanish vs Kiko, TLR4 and ITGA9 had SNPs under selection. For this analysis, the same SNP identified under selection in *ITGA9* gene in Kiko and Spanish vs Boer analysis was observed. For TLR4, 2 more SNPs in exon 4 were identified under selection. Thus, it seems that for goats, *TLR4* and *ITGA9* genes could play important roles during *H. contortus* infection.

### Common signatures of selection in sheep and goats

During the last two decades, results have shown differences in feeding behavior and gastrointestinal nematode parasitism between sheep and goats. Feeding behavior is one important aspect that differentiates sheep and goats. Sheep are typically raised in grazing systems with parasites commonly found within the pastures, and have to counteract the negative effects of GNI by developing an effective immune response. Goats are common browsers which allows them to rely less on immune response mechanisms [65].

For many years, there has been a question of the importance of immune effector molecules and the mucosal response in goats during GNI. Our findings suggest a possible interplay between Th1 and Th2 responses with conserved breed specific mechanisms. For both species, our results suggest a possible interplay between Th1 and Th2 response during GNI, as previously described by Hassan et al. [39] and Pernthaner et al. [66].

One of the most interesting findings from this study is the identification of shared immune response mechanisms between sheep and goats (Fig. 3). It is possible that some immune response mechanisms are shared between both species and induce an effective immune response against *H. contortus.* The *NOS2*, *TGFB2*, and *TLR4* genes, observed under selection in both species, are key modulators of Th1 and Th2 responses, and active players of antigen recognition. Several studies in cashmere and dairy goats have evaluated the responsiveness of resistant animals to GNI and have found a negative correlated response between worm counts and eosinophil, mast cell, and globule leucocyte counts [67–69]. In sheep, similar cellular immune response has been associated to GNI [70, 71]. Thus, it is possible that some mechanisms of immune protection are shared between these species but more studies are required to understand these events during *H. contortus* infection.

The conserved mechanisms of protective response against *H. contortus* are most likely to be useful targets in the development of alternative nematode control strategies in both species, as they can be widely applied in production systems. For this reason our future efforts will focus on validation of the results observed in the present study to unravel genetic mechanisms used for controlling *H. contortus* or other GNI between sheep and goats.

## Conclusion

Results from this study support the hypothesis that resistance to gastrointestinal parasites such as *H. contortus* is likely to be controlled by many loci. Different immune response mechanisms between sheep and goats are used to control *H. contortus* but some aspects are shared in both species. Shared mechanisms of immune protection could be useful targets in breeding programs aimed to produce resistant animals and future research is necessary to validate our findings.

## Methods

### Animal populations

The research protocol for the present study was approved by the Langston University Animal Care and Use Committee. Sire candidates were randomly selected in the first year from five commercial farms in Arkansas (CWC Farm), Kansas (Hogan Ranch), and Missouri (CMI Dorpers, Thousand Oak Ranch and Persimmon Creek Ranch) and American Institute for Goat Research at Langston University, Oklahoma and transferred to Langston University for a central sire performance test with an artificial *H. contortus* infection described later. In the second and third years, young male offspring of resistant or moderate resistant breeding groups to GIN were tested with the artificial *H. contortus* infection. Sheep and goats were grouped per breed and species in adjacent pens with automated feeders allowing free-choice access to a 15% crude protein diet at Langston University. Overall, 145 offspring sheep from Dorper (*n* = 48), Katahdin (*n* = 57), and St. Croix (*n* = 40) breeds and 144 offspring goats from Boer (*n* = 52), Kiko (*n* = 44) and Spanish (*n* = 48) breeds were used in this study.

Deworming and *H. contortus* artificial infection methods are described in a previous publication [25]. Briefly, sheep and goats were treated with albendazole (Valbazen®; 10 and 20 mg per kg of body weight, respectively) and levamisole (Prohibit®; 12 and 18 mg per kg of body weight, respectively) during 2 weeks of adaptation. Then, animals were screened for FEC reduction (< 100 epg) and received an oral dose of 10,000 L_3_ larvae of *H. contortus*. FEC was recorded at 28, 35 and 42 days post-infection. Animals returned to commercial farms at the end of the study.

### Genotyping and data quality control

Blood samples from sheep and goats were collected by puncture of the jugular vein using vacutainer tubes with anticoagulant EDTA. Subsequently, genomic DNA was isolated using DNeasy Blood & Tissue Kit (Qiagen, Valencia, CA) according the manufacturer’s instructions and stored at − 20 °C. The DNA yield was calculated from a spectrophotometric measurement at 260 nm (NanoDrop-1000, Thermo Scientific), and the purity was assessed using a ratio 260/280 nm.

Two hundred and fifty ng/μL of genomic DNA was genotyped using Capture-Sequencing by RAPiD Genomics (Gainesville, Florida) to target 100 genes related to the immune response against *H. contortus* or other GNI. The candidate gene panel was selected for targeted sequencing based on results from previous studies in sheep [55, 72–76] and goats [77]. In addition, genes related to the immune response against *H. contortus* and other GNI were considered as candidates for targeted sequencing (Additional file 6: Table S5).

The Oar_v4.0 reference genome available at the National Center for Biotechnology Information (NCBI) genome browser was used to design biotinylated 120-mer probes that captured sequences at each target locus. For library preparation, Nextera tagmentation protocol from Illumina was used. Then, biotin-labeled probes hybridized denatured libraries and streptavidin-coated beads were used to capture the probe-library complex. Streptavidin-coated beads were magnetically pulled down and DNA fragments were eluted. Libraries were captured by complimentary surface bound oligos and library amplification was performed using bridge amplification according to Illumina’s guidelines. The probe set used for sequence capture contained 5000 probes representing 100 genes. Target enriched libraries were sequenced using the Illumina HiSeq 3000 PE100 platform to generate 2 × 101 bp paired-end reads.

Data was demultiplexed using bcl2fastq conversion software from Illumina, cleaned, and trimmed. The 3′ ends were trimmed and low quality bases with < 20 Phred quality score reads were removed. Clean reads were mapped to the sheep (Oar_v4.0) and goat (ASM170441v1) reference genomes with MOSAIK software [78]. Freebayes was used for identification of SNPs and VCFtools [79] was used to generate VCF files. Samples were filtered based on maximum missing count [3], minimum number of alleles [2], mean read depth (750), call rate (< 95%) and MAF (≤ 0.01). Thus, SNPs with a call rate < 95% and MAF ≤ 0.01 were removed.

Principal component analysis plots were generated to illustrate population structure using JMP Genomics 9 software from SAS (SAS Institute Inc., Cary, NC). Individuals included in the principal component analysis and further *F*st analysis were selected based on the identity by state threshold of ≤0.5. For these analyses, one hundred and twenty sheep from Dorper (*n* = 40), Katahdin (*n* = 40), and St. Croix (*n* = 40) breeds and 129 goats from Boer (*n* = 43), Kiko (*n* = 43) and Spanish (*n* = 43) breeds were used.

### Identification of signatures of selection using allele frequencies

Signatures of selection were identified using *F*st statistic at each SNP using frequentist and Bayesian approaches which are focused on the identification of differences in allele frequencies between subpopulations. To identify genetic divergence between subpopulations, analyses between resistant and susceptible breeds within species were carried out. St. Croix and Katahdin were considered resistant sheep breeds and were compared against Dorper which was considered the susceptible sheep breed. Analyses were performed as follow: Katahdin and St. Croix vs Dorper (KS vs D, pooled resistant breeds vs susceptible breed), Katahdin vs Dorper (K vs D, resistant vs susceptible breed), and St. Croix vs Dorper breeds (S vs D, resistant vs susceptible breed). Similarly, for goats, Spanish and Kiko breeds were classified as resistant and compared against the susceptible Boer breed. Identification of signatures of selection in goats between resistant and susceptible breeds were performed as follows: Kiko and Spanish vs Boer (KS vs B, pooled resistant breeds vs susceptible breed), Kiko vs Boer (K vs B, resistant vs susceptible breed), and Spanish vs Boer (S vs B, resistant vs susceptible).

To identify any signatures of selection different in the two resistant breeds, an additional analysis was performed per species by comparing the St. Croix against Katahdin (S vs K, resistant vs resistant breed) for sheep, and Spanish against Kiko (S vs K, resistant vs resistant breed) for goats.

For the frequentist *F*st, calculation of average allele frequency across breeds, estimation of total variance, and deviation of each population from mean and *F*st computation were performed using the R software and the R codes from Gondro et al. [22]. The formula used to estimate *F*st values was the following:

$$ Fst=\frac{{\left( deviation\ of\ each\ population\ from\ mean\right)}^2}{total\ variance}=\frac{\sigma^2 subpopulation}{\sigma^2 total} $$

where σ^2^subpopulation is the variance of the deviation of each population from mean and σ^2^total is the total variance. Estimates corresponding to the 1% extreme *F*st values were used to define a significance threshold and identify regions under selection.

Bayesian *F*st was estimated using BayeScan software. In this approach, a Bayesian likelihood method implemented via reversible jump Markov Chain Monte Carlo (MCMC) was used which assumes that allele frequencies follow a Dirichlet distribution [80]. The main advantage of this approach is that *F*st statistic is modelled using logistic regression methods by decomposing locus–population *F*st coefficients into a population-specific component (beta), shared by all loci and a locus-specific component (alpha) shared by all the populations. Then, departure from neutrality at a given locus is assumed when the locus-specific component is necessary to explain the observed pattern of diversity (alpha significantly different from 0). Diversifying selection can be assumed if positive values of alpha are observed, whereas negative alpha values suggest balancing or purifying selection. Consequently, two alternative models are generated for each locus, including or not the alpha component to model selection. BayeScan software uses a reversible-jump MCMC algorithm to estimate the posterior probability of these models [80–82]. For the Markov chain Monte Carlo algorithm implemented in BayeScan, 20 pilot runs of 5000 iterations were used to adjust the proposal distribution to have acceptance rates between 0.25 and 0.45 for the runs. Then, a burn-in of 10,000 iterations followed by 100,000 iterations were used for estimation [80–82].

Candidate SNPs under selection were located using the sheep (Oar_v4.0) and goat (ASM170441v1) reference genomes from NCBI. The Online Mendelian Inheritance in Man website and scientific literature were used to determine gene function.

## Supplementary information

**Additional file 1: Figure S1.** Manhattan plots of the frequentist *F*st values per SNP from targeted genomic regions in sheep and goats. The SNP data is ordered based on chromosomal location (x-axis). For sheep, plots includes: Katahdin and St. Croix vs Dorper (KS vs D, pooled resistant breeds vs susceptible breed), Katahdin vs Dorper (K vs D, resistant vs susceptible breed), St. Croix vs Dorper (S vs D, resistant vs susceptible breed) and St. Croix vs Katahdin (S vs K, resistant vs resistant breed) analyses. For goats, plots includes: Kiko and Spanish vs Boer (KS vs B, pooled resistant breeds vs susceptible breed), Kiko vs Boer (K vs B, resistant vs susceptible breed), Spanish vs Boer (S vs B, resistant vs susceptible breed) and Spanish vs Kiko (S vs K, resistant vs resistant breed) analyses. The genes with SNPs under selection in each analysis are presented in a box on the right side of Manhattan plots.

**Additional file 2: Table S1.** Signatures of selection identified between resistant (Katahdin or St. Croix) and susceptible (Dorper) sheep breeds using frequentist *F*st. Breeds compared (comparison), gene name, gene region, SNP name (chromosome and position), SNP, mutation type (synonymous or missense), and *F*st value for the SNPs under selection.

**Additional file 3: Table S2.** Signatures of selection identified between resistant (Katahdin or St. Croix) and susceptible (Dorper) sheep breeds using Bayesian *F*st. Breeds compared (comparison), gene name, gene region, SNP name (chromosome and position), SNP, mutation type (synonymous or missense), and *F*st value for the SNPs under selection.

**Additional file 4: Table S3.** Signatures of selection identified between resistant (Kiko or Spanish) and susceptible (Boer) goat breeds using frequentist *F*st. Breeds compared (comparison), gene name, gene region, SNP name (chromosome and position), SNP, mutation type (synonymous or missense), and *F*st value for the SNPs under selection.

**Additional file 5: Table S4.** Signatures of selection identified between resistant (Kiko or Spanish) and susceptible (Boer) goat breeds using Bayesian *F*st. Breeds compared (comparison), gene name, gene region, SNP name (chromosome and position), SNP, mutation type (synonymous or missense), and *F*st value for the SNPs under selection.

**Additional file 6: Table S5.** Gene table

## Data Availability

The datasets generated during and/or analyzed during the current study are available on NCBI BioProject (https://www.ncbi.nlm.nih.gov/bioproject), accession number PRJEB32310 (sheep) and PRJEB32312 (goat).

## Abbreviations

CHR: Chromosome number in goat
FEC: Fecal egg count
GIN: Gastrointestinal nematodes
OAR: Chromosome number in sheep
