# Supplementary figures and images for: Signatures of selection for resistance to Haemonchus contortus in sheep and goats
Source: BMC Genomics. 2019 Oct 15;20:735. doi: 10.1186/s12864-019-6150-y (PMC6792194; doi:10.1186/s12864-019-6150-y)

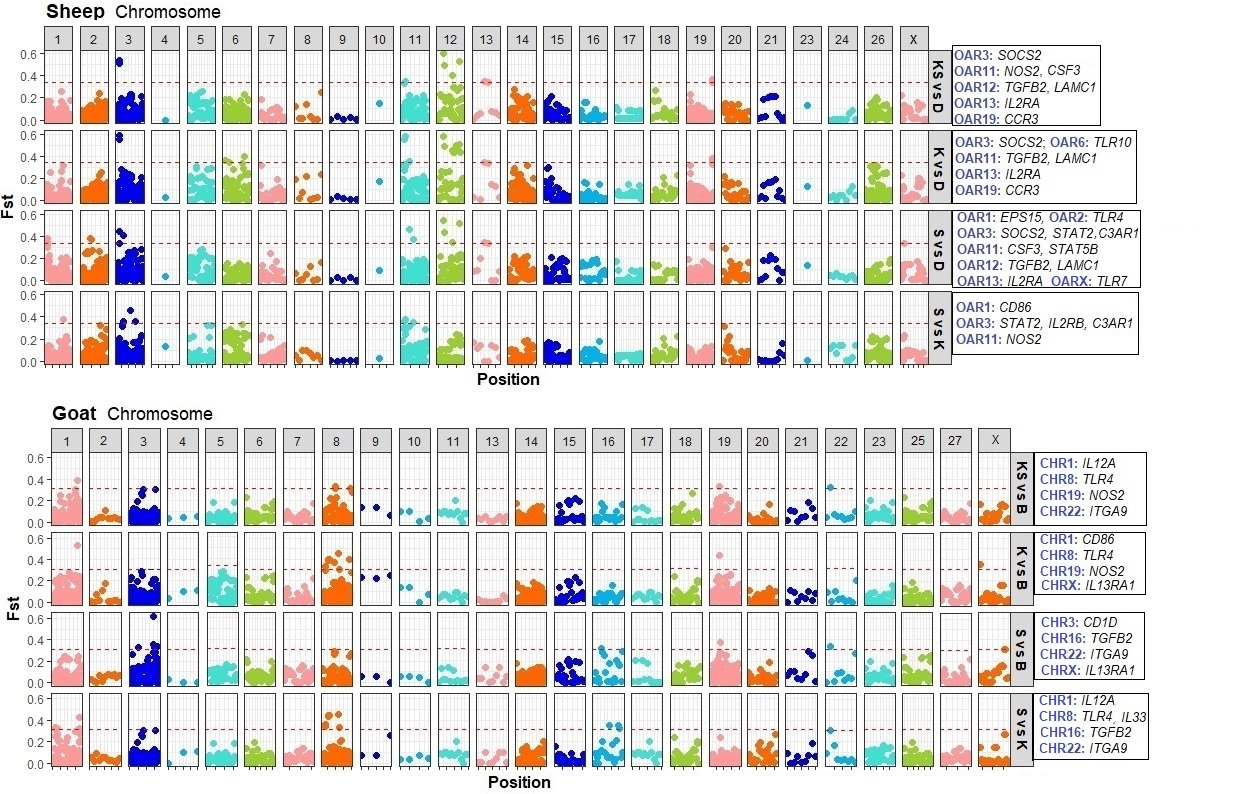

Supplement: Supplementary file 1 — Additional file 1: Figure S1. Manhattan plots of the frequentist Fst values per SNP from targeted genomic regions in sheep and goats. The SNP data is ordered based on chromosomal location (x-axis). For sheep, plots includes: Katahdin and St. Croix vs Dorper (KS vs D, pooled resistant breeds vs susceptible breed), Katahdin vs Dorper (K vs D, resistant vs susceptible breed), St. Croix vs Dorper (S vs D, resistant vs susceptible breed) and St. Croix vs Katahdin (S vs K, resistant vs resistant breed) analyses. For goats, plots includes: Kiko and Spanish vs Boer (KS vs B, pooled resistant breeds vs susceptible breed), Kiko vs Boer (K vs B, resistant vs susceptible breed), Spanish vs Boer (S vs B, resistant vs susceptible breed) and Spanish vs Kiko (S vs K, resistant vs resistant breed) analyses. The genes with SNPs under selection in each analysis are presented in a box on the right side of Manhattan plots. [file 12864_2019_6150_MOESM1_ESM.jpg]
