# Supplementary material for: Signatures of selection for resistance to Haemonchus contortus in sheep and goats
Source: BMC Genomics. 2019 Oct 15;20:735. doi: 10.1186/s12864-019-6150-y (PMC6792194; doi:10.1186/s12864-019-6150-y)
Supplement: Supplementary file 2 — Additional file 2: Table S1. Signatures of selection identified between resistant (Katahdin or St. Croix) and susceptible (Dorper) sheep breeds using frequentist Fst. Breeds compared (comparison), gene name, gene region, SNP name (chromosome and position), SNP, mutation type (synonymous or missense), and Fst value for the SNPs under selection. [file 12864_2019_6150_MOESM2_ESM.docx]

Additional file 2: **Table S1.** Signatures of selection identified between resistant (Katahdin or St. Croix) and susceptible (Dorper) sheep breeds using frequentist *F*st. Breeds compared (comparison), gene name, gene region, SNP name (chromosome and position), SNP, mutation type (synonymous or missense), and *F*st value for the SNPs under selection.

| **Comparison** | **Gene** | **Region** | **SNP name** | **SNP** | **Mutation** | ***F*st** |
| --- | --- | --- | --- | --- | --- | --- |
| **Katahdin and St. Croix vs Dorper** (Resistant and Resistant vs Susceptible) | SOCS2 | 3' UTR | OAR3:129558430 | G/A |  | 0.51 |
|  | NOS2 | Exon 7 | OAR11:18963484 | A/G | Synonymous (Ile → Ile) | 0.35 |
|  | NOS2 | Exon 16 | OAR11:18963494 | T/C | Synonymous (Leu → Leu) | 0.35 |
|  | CSF3 | 5'UTR | OAR11:39857496 | G/A |  | 0.35 |
|  | TGFB2 | 3'UTR | OAR12:19965761 | A/C |  | 0.57 |
|  | TGFB2 | 3'UTR | OAR12:19965865 | A/C |  | 0.51 |
|  | LAMC1 | Intron 19 | OAR12:62193113 | T/C |  | 0.52 |
|  | LAMC1 | 3'UTR | OAR12:62208066 | G/A |  | 0.41 |
|  | IL2RA | Intron 5 | OAR13:10442920 | C/A |  | 0.21 |
|  | IL2RA | Intron 5 | OAR13:10442953 | A/G |  | 0.21 |
|  | CCR3 | Exon 1 | OAR19:53067498 | A/G | Missense (Leul → Gly ) | 0.22 |
| **Katahdin vs Dorper** (Resistant vs Susceptible) | SOCS2 | Exon 2 | OAR3:129558034 | C/T | Synonymous (Ile → Ile) | 0.6 |
|  | SOCS2 | 3' UTR | OAR3:129558430 | G/A |  | 0.56 |
|  | TLR10 | Exon 2 | OAR6:57923205 | G/C | Missense (Val → Leu) | 0.421 |
|  | NOS2 | Exon 7 | OAR11:18963484 | A/G | Synonymous (Ile → Ile) | 0.59 |
|  | NOS2 | Exon 16 | OAR11:18963494 | T/C | Synonymous (Leu → Leu) | 0.56 |
|  | TGFB2 | 3'UTR | OAR12:19965761 | A/C |  | 0.57 |
|  | TGFB2 | 3'UTR | OAR12:19965865 | A/C |  | 0.51 |
|  | LAMC1 | Intron 19 | OAR12:62193113 | T/C |  | 0.52 |
|  | LAMC1 | 3'UTR | OAR12:62208066 | G/A |  | 0.41 |
|  | IL2RA | Intron 5 | OAR13:10442920 | C/A |  | 0.21 |
|  | IL2RA | Intron 5 | OAR13:10442953 | A/G |  | 0.22 |
| **St. Croix vs Dorper** (Resistant vs Susceptible) | EPS15 | 3'UTR | OAR1:25516496 | G/A |  | 0.35 |
|  | TLR4 | Exon 4 | OAR2:5882565 | A/G | Missense (Phe → Leu) | 0.37 |
|  | SOCS2 | Exon 2 | OAR3:129558034 | C/T | Synonymous (Ile → Ile) | 0.41 |
|  | SOCS2 | 3' UTR | OAR3:129558430 | G/A |  | 0.42 |
|  | C3AR1 | 3'UTR | OAR3:206099209 | T/A |  | 0.35 |
|  | NOS2 | Exon 7 | OAR11:18963484 | A/G | Synonymous (Ile → Ile) | 0.39 |
|  | NOS2 | Exon 16 | OAR11:18963494 | T/C | Synonymous (Leu → Leu) | 0.48 |
|  | STAT5B | Intron 16 | OAR11:41755713 | G/A |  | 0.39 |
|  | CSF3 | 5'UTR | OAR11:39857496 | G/A |  | 0.48 |
|  | TGFB2 | 3'UTR | OAR12:19965761 | A/C |  | 0.55 |
|  | TGFB2 | 3'UTR | OAR12:19965865 | A/C |  | 0.47 |
|  | LAMC1 | Intron 19 | OAR12:62193113 | T/C |  | 0.52 |
|  | IL2RA | Intron 5 | OAR13:10442920 | C/A |  | 0.21 |
|  | IL2RA | Intron 5 | OAR13:10442953 | A/G |  | 0.22 |
|  | TLR7 | Exon 1 | OARX:10367470 | G/A | Synonymous (Leu → Leu) | 0.21 |
| **St. Croix vs Katahdin** (Resistant vs Resistant) | CD86 | 3'UTR | OAR1:184659051 | C/T |  | 0.38 |
|  | STAT2 | Exon 20 | OAR3:162724229 | A/G | Missense (His → Arg) | 0.35 |
|  | IL2RB | Exon 10 | OAR3:180152504 | T/C | Missense ( Ile →Val) | 0.45 |
|  | C3AR1 | 3'UTR | OAR3:206099209 | T/A |  | 0.36 |
|  | NOS2 | Exon 6 | OAR11:18975484 | T/C | Synonymous (Leu → Leu) | 0.36 |
|  | NOS2 | Exon 16 | OAR11:18963494 | A/G | Synonymous (Ile → Ile) | 0.37 |
