# Supplementary material for: Signatures of selection for resistance to Haemonchus contortus in sheep and goats
Source: BMC Genomics. 2019 Oct 15;20:735. doi: 10.1186/s12864-019-6150-y (PMC6792194; doi:10.1186/s12864-019-6150-y)
Supplement: Supplementary file 3 — Additional file 3: Table S2. Signatures of selection identified between resistant (Katahdin or St. Croix) and susceptible (Dorper) sheep breeds using Bayesian Fst. Breeds compared (comparison), gene name, gene region, SNP name (chromosome and position), SNP, mutation type (synonymous or missense), and Fst value for the SNPs under selection. [file 12864_2019_6150_MOESM3_ESM.docx]

Additional file 3: **Table S2.** Signatures of selection identified between resistant (Katahdin or St. Croix) and susceptible (Dorper) sheep breeds using Bayesian *F*st. Breeds compared (comparison), gene name, gene region, SNP name (chromosome and position), SNP, mutation type (synonymous or missense), and *F*st value for the SNPs under selection.

| **Comparison** | **Gene** | **Region** | **SNP name** | **SNP** | **MAF** | **Mutation** | ***F*st** |
| --- | --- | --- | --- | --- | --- | --- | --- |
| **Katahdin and St. Croix vs Dorper** (Resistant and Resistant vs Susceptible) | CSF3 | 5'UTR | OAR11:39857496 | G/A | Dorper: 0.20, Katahdin: 0.19, Spanish: 0.35 |  | 0.31 |
|  | NOS2 | Exon 7 | OAR11:18963484 | A/G | Dorper: 0.21, Katahdin: 0.19, Spanish: 0.23 | Synonymous (Ile → Ile) | 0.36 |
|  | NOS2 | Exon 16 | OAR11:18963494 | T/C | Dorper: 0.20, Katahdin: 0.19, Spanish: 0.25 | Synonymous (Leu → Leu) | 0.33 |
|  | STAT5B | Intron 16 | OAR11:41755713 | G/A | Dorper: 0.20, Katahdin: 0.18, Spanish: 0.48 |  | 0.28 |
|  | TGFB2 | 3'UTR | OAR12:19965761 | A/C | Dorper: 0.20, Katahdin: 0.12, Spanish: 0.16 |  | 0.26 |
|  | IL2RA | Intron 5 | OAR13:10442920 | C/A | Dorper: 0.55, Katahdin: 0, Spanish: 0 |  | 0.24 |
|  | IL2RA | Intron 5 | OAR13:10442953 | A/G | Dorper: 0.43, Katahdin: 0, Spanish: 0 |  | 0.23 |
| **Katahdin vs Dorper** (Resistant vs Susceptible) | SOCS2 | Exon 2 | OAR3:129558034 | C/T | Dorper: 0.20, Katahdin: 0.19 | Synonymous (Ile → Ile) | 0.25 |
|  | SOCS2 | 3' UTR | OAR3:129558430 | G/A | Dorper: 0.20, Katahdin: 0.19 |  | 0.21 |
|  | NOS2 | Exon 7 | OAR11:18963484 | A/G | Dorper: 0.21, Katahdin: 0.19 | Synonymous (Ile → Ile) | 0.36 |
|  | NOS2 | Exon 16 | OAR11:18963494 | T/C | Dorper: 0.20, Katahdin: 0.19 | Synonymous (Leu → Leu) | 0.33 |
|  | TGFB2 | 3'UTR | OAR12:19965761 | A/C | Dorper: 0.20, Katahdin: 0.12 |  | 0.26 |
|  | TGFB2 | 3'UTR | OAR12:19965865 | A/C | Dorper: 0.05, Katahdin: 0.29 |  | 0.25 |
|  | IL2RA | Intron 5 | OAR13:10442920 | C/A | Dorper: 0.55, Katahdin: 0 |  | 0.24 |
|  | IL2RA | Intron 5 | OAR13:10442953 | A/G | Dorper: 0.43, Katahdin: 0 |  | 0.23 |
| **St. Croix vs Dorper** (Resistant vs Susceptible) | CSF3 | 5'UTR | OAR11:39857496 | G/A | Dorper: 0.20, Spanish: 0.35 |  | 0.34 |
|  | STAT5B | Intron 16 | OAR11:41755713 | G/A | Dorper: 0.20, Spanish: 0.48 |  | 0.28 |
|  | TGFB2 | 3'UTR | OAR12:19965761 | A/C | Dorper: 0.20, Spanish: 0.16 |  | 0.23 |
|  | IL2RA | Intron 5 | OAR13:10442920 | C/A | Dorper: 0.55, Spanish: 0 |  | 0.21 |
|  | TLR7 | Exon 1 | OARX:10367470 | G/A | Dorper: 0, Spanish: 0.28 | Synonymous (Leu → Leu) | 0.26 |
|  | LTBR | Intron 8 | OAR3:207705264 | G/C | Dorper: 0, Spanish: 0.32 |  | 0.24 |
|  | C3AR1 | 3'UTR | OAR3:206099209 | T/A | Dorper: 0, Spanish: 0.42 |  | 0.21 |
|  | C3AR1 | 3'UTR | OAR3:206099221 | A/C | Dorper: 0, Spanish: 0.37 |  | 0.21 |
