# Supplementary material for: Signatures of selection for resistance to Haemonchus contortus in sheep and goats
Source: BMC Genomics. 2019 Oct 15;20:735. doi: 10.1186/s12864-019-6150-y (PMC6792194; doi:10.1186/s12864-019-6150-y)
Supplement: Supplementary file 4 — Additional file 4: Table S3. Signatures of selection identified between resistant (Kiko or Spanish) and susceptible (Boer) goat breeds using frequentist Fst. Breeds compared (comparison), gene name, gene region, SNP name (chromosome and position), SNP, mutation type (synonymous or missense), and Fst value for the SNPs under selection. [file 12864_2019_6150_MOESM4_ESM.docx]

Additional file 4: **Table S3.** Signatures of selection identified between resistant (Kiko or Spanish) and susceptible (Boer) goat breeds using frequentist *F*st. Breeds compared (comparison), gene name, gene region, SNP name (chromosome and position), SNP, mutation type (synonymous or missense), and *F*st value for the SNPs under selection.

| **Comparison** | **Gene** | **Region** | **SNP name** | **SNP** | **Mutation** | ***F*st** |
| --- | --- | --- | --- | --- | --- | --- |
| **Kiko and Spanish vs Boer** (Resistant vs Susceptible) | IL12A | 5'UTR | CHR1: 106973801 | A/G |  | 0.4 |
|  | TLR4 | Exon 3 | CHR8:106725462 | T/C | Synonymous (Ser → Ser) | 0.36 |
|  | TLR4 | Exon 3 | CHR8:106725265 | A/G | Synonymous (Leu → Leu) | 0.35 |
|  | NOS2 | Intron 2 | CHR19:19239245 | A/T |  | 0.37 |
|  | ITGA9 | 3'UTR | CHR22:11106216 | A/T |  | 0.36 |
| **Kiko vs Boer** (Resistant vs Susceptible) | CD86 | Intron 1 | CHR1:66217253 | C/T |  | 0.53 |
|  | TLR4 | Exon 3 | CHR8:106725462 | T/C | Synonymous (Ser → Ser) | 0.4 |
|  | TLR4 | Exon 3 | CHR8:106725265 | A/G | Synonymous (Leu → Leu) | 0.43 |
|  | TLR4 | Exon 4 | CHR8:106725156 | G/A | Synonymous (His → His) | 0.42 |
|  | TLR4 | Exon 4 | CHR8:106725045 | C/T | Synonymous (Leu → Leu) |  |
|  | IL33 | Exon 10 | CHR8:38344904 | A/G | Synonymous (Thr → Thr) | 0.44 |
|  | NOS2 | Intron 2 | CHR19:19239245 | A/T |  | 0.48 |
|  | IL13RA1 | 3'UTR | CHR22:25115674 | G/T |  | 0.4 |
| **Spanish vs Boer** (Resistant vs Susceptible) | CD1D | Exon 2 | CHR3:107890049 | T/G | Synonymous (Ser → Ser) | 0.61 |
|  | TGFB2 | 3'UTR | CHR16:20438403 | T/G |  | 0.35 |
|  | NOS2 | Intron 2 | CHR19:19239245 | A/T |  | 0.38 |
|  | ITGA9 | 3'UTR | CHR22:11106216 | A/T |  | 0.37 |
|  | IL13RA1 | 3'UTR | CHRX:25115674 | G/T |  | 0.35 |
| **Spanish vs Kiko** (Resistant vs Resistant) | IL12A | 5'UTR | CHR1: 106973801 | A/G |  | 0.4 |
|  | TLR4 | Exon 3 | CHR8:106725265 | A/G | Synonymous (Leu → Leu) | 0.39 |
|  | TLR4 | Exon 4 | CHR8:106725156 | G/A | Synonymous (His → His) | 0.41 |
|  | TLR4 | Exon 4 | CHR8:106725045 | C/T | Synonymous (Leu → Leu) | 0.42 |
|  | IL33 | Exon 10 | CHR8:38344904 | A/G | Synonymous (Thr → Thr) | 0.36 |
|  | TGFB2 | 3'UTR | CHR16:20438403 | T/G |  | 0.38 |
|  | ITGA9 | 3'UTR | CHR22:11106216 | A/T |  | 0.35 |
