# Supplementary material for: Signatures of selection for resistance to Haemonchus contortus in sheep and goats
Source: BMC Genomics. 2019 Oct 15;20:735. doi: 10.1186/s12864-019-6150-y (PMC6792194; doi:10.1186/s12864-019-6150-y)
Supplement: Supplementary file 5 — Additional file 5: Table S4. Signatures of selection identified between resistant (Kiko or Spanish) and susceptible (Boer) goat breeds using Bayesian Fst. Breeds compared (comparison), gene name, gene region, SNP name (chromosome and position), SNP, mutation type (synonymous or missense), and Fst value for the SNPs under selection. [file 12864_2019_6150_MOESM5_ESM.docx]

Additional file 5: **Table S4.** Signatures of selection identified between resistant (Kiko or Spanish) and susceptible (Boer) goat breeds using Bayesian *F*st. Breeds compared (comparison), gene name, gene region, SNP name (chromosome and position), SNP, mutation type (synonymous or missense), and *F*st value for the SNPs under selection.

| **Comparison** | **Gene** | **Region** | **SNP name** | **SNP** | **MAF across breeds** | **Mutation** | ***F*st** |
| --- | --- | --- | --- | --- | --- | --- | --- |
| **Kiko and Spanish vs Boer** (Resistant vs Susceptible) | IL12A | 5'UTR | CHR1: 106973801 | A/G | Boer: 0, Kiko: 0.3, Spanish:0 |  | 0.23 |
|  | TLR4 | Exon 3 | CHR8:106725462 | T/C | Boer: 0.05, Kiko: 0.36, Spanish: 0 | Synonymous (Ser → Ser) | 0.30 |
|  | TLR4 | Exon 3 | CHR8:106725265 | A/G | Boer: 0.05, Kiko: 0.3, Spanish: 0 | Synonymous (Leu → Leu) | 0.28 |
|  | TLR4 | Exon 4 | CHR8:106725156 | G/A | Boer: 0.05, Kiko: 0.32, Spanish:0 | Synonymous (His → His) | 0.29 |
|  | TLR4 | Exon 4 | CHR8:106725045 | C/T | Boer: 0.05, Kiko: 0.34, Spanish:0 | Synonymous (Leu → Leu) | 0.29 |
|  | IL33 | Exon 10 | CHR8:38344904 | A/G | Boer: 0.24, Kiko: 0.08, Spanish: 0.32 | Synonymous (Thr → Thr) | 0.22 |
|  | TGFB2 | 3'UTR | CHR16:20438403 | T/G | Boer: 0, Kiko: 0.13, Spanish: 0.38 |  | 0.31 |
|  | ITGA9 | 3'UTR | CHR22:11106216 | A/T | Boer: 0.48, Kiko: 0.42, Spanish: 0 |  | 0.28 |
| **Kiko vs Boer** (Resistant vs Susceptible) | CD86 | Intron 1 | CHR1:66217253 | C/T | Boer: 0, Kiko: 0.15 |  | 0.21 |
| **Spanish vs Boer** (Resistant vs Susceptible) | CD1D | Exon 2 | CHR3:107890049 | T/G | Boer: 0.20 , Spanish: 0.28 | Synonymous (Ser → Ser) | 0.27 |
|  | TGFB2 | 3'UTR | CHR16:20438403 | T/G | Boer: 0, Spanish: 0.38 |  | 0.35 |
|  | ITGA9 | 3'UTR | CHR22:11106216 | A/T | Boer: 0.48, Spanish: 0 |  | 0.21 |
|  | IL13RA1 | 3'UTR | CHRX:25115674 | G/T | Boer: 0, Spanish: 0.27 |  | 0.22 |
| **Spanish vs Kiko** (Resistant vs Resistant) | TLR4 | Exon 3 | CHR8:106725462 | T/C | Kiko: 0.36, Spanish: 0 | Synonymous (Ser → Ser) | 0.3 |
|  | TLR4 | Exon 3 | CHR8:106725265 | A/G | Kiko: 0.3, Spanish: 0 | Synonymous (Leu → Leu) | 0.29 |
|  | TLR4 | Exon 4 | CHR8:106725156 | G/A | Kiko: 0.32, Spanish:0 | Synonymous (His → His) | 0.29 |
|  | TLR4 | Exon 4 | CHR8:106725045 | C/T | Kiko: 0.34, Spanish:0 | Synonymous (Leu → Leu) | 0.29 |
|  | ITGA9 | 3'UTR | CHR22:11106216 | A/T | Kiko: 0.42, Spanish: 0 |  | 0.32 |
